# Development of a Prandtl-Ishlinskii hysteresis model for a large capacity magnetorheological fluid damper

**DOI:** 10.1177/10775463241248963

**Published:** 2024-05-06

**Authors:** Hossein Vatandoost, Moustafa Abdalaziz, Ramin Sedaghati, Subhash Rakheja

**Affiliations:** Department of Mechanical, Industrial and Aerospace Engineering, 5618 Concordia University, Montreal, QC, Canada

**Keywords:** Magnetorheological fluid damper, variable damping and stiffness, hysteresis modeling, Prandtl-Ishlinskii model, friction element, dynamic characterization

## Abstract

Magnetorheological (MR) fluid (MRF) dampers, serving as fail-safe semi-active devices, exhibit nonlinear hysteresis characteristics, emphasizing the necessity for accurate modeling to formulate effective control strategies in smart systems. This paper introduces a novel stop operator-based Prandtl-Ishlinskii (PI) model, featuring a reduced parameter set (seven), designed to estimate the nonlinear hysteresis properties of a large-scale bypass MRF damper with variable stiffness capabilities under varying applied current. With only seven parameters, the model realizes current, displacement, and rate dependencies. The force-displacement and force-velocity responses of the designed MRF damper were experimentally characterized under broad ranges of applied current (0–2 A), excitation frequency (0.5–4 Hz), and displacement amplitude (1–2.5 mm). A training dataset was subsequently used to develop a novel field-dependent modified PI model, incorporating multiple hysteresis operators with and without a friction element. The proposed model accurately predicted the MRF damper behavior within the training dataset, and its validity was assessed against data from diverse experimental conditions. The PI model with friction element generally outperformed the model without friction when frequency exceeds 0.5 Hz, demonstrating its ability to characterize nonlinear hysteresis force-displacement and force-velocity properties of the MRF damper under the ranges of applied current and excitations considered with reasonable accuracy. Experimental data were also estimated by the Bouc–Wen model, and compared with those obtained via the formulated PI model, affirming the overall superiority of the proposed PI models considering the computational cost, and total number of parameters. Leveraging the simplicity, minimal parameter requirements, and analytic invertibility of PI models, the proposed PI model is considered a superior choice for modeling and subsequently controlling smart structures employing MRF dampers.

## 1. Introduction

Ground vehicles can cause a variety of negative effects due to vibration, which can range from discomfort or annoyance to severe degeneration of the spine and supporting structures (Rakheja et al., 2020). In particular, the perception of whole-body vibration (WBV) induced by road conditions is a significant concern for human occupants of vehicles, as it can increase the risk of motion sickness in addition to discomfort and annoyance (Diels et al., 2016). Exposure to WBV, combined with extended periods of seating in a confined environment, often involving twisted head and neck positions, has been linked to multiple adverse health effects on human biomechanics and physiological responses. These effects include musculoskeletal issues in the spine and head/neck, along with fatigue and motion sickness (Griffin and Erdreich, 1991). Significant progress has been made in the development of semi-active suspensions, such as magnetorheological fluid (MRF) dampers, which aim to reduce the transmission of WBV to vehicle occupants (Ding et al., 2023; Heidarian and Wang, 2019; Sun et al., 2016; Wang et al., 2023).

Significant work has been dedicated to modeling MRF dampers in order to predict their force-displacement and force-velocity hysteresis characteristics under different levels of mechanical excitation and applied currents. These models are essential for advancing the developments in control strategies for active or semi-active vibration control devices based on smart MRF dampers. The reported models may be classified into quasi-static and dynamic models. The vast majority of the studies have focused on quasi-static models considering Bigham plastic (BP) and Herschel–Buckley (HB) behavior of MR fluids (Chooi and Oyadiji, 2009; Wereley and Pang, 1998). Although such models have provided considerable knowledge for early stages of design, these do not provide dynamic force-displacement and force-velocity characteristics, which invariably exhibit considerable hysteresis.

Dynamic models for MRF dampers have typically utilized either physics-based or phenomenological-based approaches to predict their force-displacement and force-velocity characteristics in relation to applied current and excitation. Phenomenological models are generally preferred over physics-based models, as the latter often rely on significant simplifying assumptions (Spencer et al., 1997). Phenomenological models also offer computational efficiency compared to physical models, with shorter computation times; however, they lack the ability to provide insight or descriptions of the corresponding physical processes (Al Saaideh et al., 2021). Phenomenological models seek to portray hysteresis using mathematical expressions, often in the form of differential equations or operators. In particular, the models that encompass differential equation include the Dahl model (Majdoub et al., 2018; Rasmussen et al., 2023), the Duhem model (Chen et al., 2018; Qian et al., 2018), and the Bouc–Wen model (Chen et al., 2022a; Dominguez et al., 2004; Jiang et al., 2021; Talatahari et al., 2012). The operator-based models include the Preisach model (Han et al., 2007; Seong et al., 2009), Prandtl-Ishlinkii (PI) model (Al Janaideh et al., 2011; Wang et al., 2011), the Krasnoselskii–Pokrovskii model (Li and Zhang, 2019; Zhang et al., 2023), and the Maxwell-Slip-based hysteresis model (Chen et al., 2022b; Madsen et al., 2021).

In terms of simplicity and parameter requirements, operator-based models generally outperform differential equation-based models. Many reported studies (for instance, (Al Janaideh et al., 2016)) have demonstrated the straightforward identification of PI model parameters, requiring only a few play operators for implementation. However, the Bouc–Wen model, relying on differential equations, typically demands seven parameters (c₀, k₀, α, A, β, γ, n) for each loading condition in modeling MRF damper behavior, requiring extensive computational efforts for hysteresis characterization (Spencer et al., 1997). Moreover, the Bouc–Wen model lacks a unique representation of input-output characteristics, posing challenges for parameter determination through data-driven identification procedures. To address this limitation, certain Bouc–Wen parameters are often set to arbitrary values (Ikhouane and Rodellar, 2007), complicating the development of rate-dependent or current/voltage-dependent models crucial for control algorithms in smart MRF devices.

Among operator-based models, the PI model, comprising weighted play operators with a density function parameterized by a single threshold variable, stands out for its simplicity, flexibility, and adaptability in handling unsaturated and saturated hysteresis nonlinearities (Al Janaideh et al., 2011, 2023; Dargahi et al., 2019). Notably, compared to Krasnoselskii–Pokrovskii and Preisach models, the output of PI models is not restricted to the interval from −1 to 1 (Minorowicz et al., 2018). Besides, the primary benefit of the PI model compared to the Preisach model lies in its ability to achieve an analytical inverse, allowing for its utilization as a feedforward compensator for the control of various smart actuators under ideal conditions (Al Janaideh et al., 2008).

Furthermore, the PI model allows for the modification of both the density function and the play or stop operator, enhancing its capability to depict hysteresis (Al Janaideh et al., 2008). This flexibility is crucial for predicting the hysteresis behavior of MRF dampers. The PI model also permits analytical inverses under mild assumptions, enabling the efficient integration of feedforward inverse compensation (Al Janaideh et al., 2023). This feature is instrumental in mitigating the impact of hysteresis, underscoring the significance of the PI model in capturing dynamic hysteretic characteristics of MRF dampers for real-time control in applications like semi-active car seat suspension systems.

Furthermore, the operator-based hysteresis models have been also widely used to describe nonlinear hysteresis phenomena in piezoelectric and magnetostrictive actuators, and pneumatic artificial muscles (Janaideh et al., 2009; Xie et al., 2018). Only a few studies, however, have explored such models for characterizing nonlinear hysteresis of MRF dampers. For example, Wang et al. developed a generalized hysteresis PI model for predicting dynamic behavior of a MRF damper under specific loading conditions (Wang et al., 2011). They used a hyperbolic in the modified PI operator to create the characteristic hysteresis curve, resembling an “S” shape, commonly observed in the force-velocity profiles of MR dampers. Their PI model involved 14 parameters for a given applied current. Identification of a field-dependent dynamic model involving fewer model parameters is, however, indispensably desirable to facilitate controller design for active or semi-active vibration control devices based on MRF dampers.

By reviewing the existing literature, it can be inferred that modeling of MRF damper concerns with challenges mainly due to their nonlinear and hysteresis force-displacement and force-velocity characteristics. These contributed to the development of quite complex models (i.e., Bouc–Wen) with large number of parameters for predicting their hysteresis behavior, making the development of their control strategies more difficult. Besides, modeling of MRF damper characteristics has rarely been investigated via PI model, particularly with current dependency and displacement, and rate dependencies. Therefore, this paper makes several key contributions, which can be summarized as follows:

• Introducing a novel stop operator-based PI model designed for estimating the nonlinear hysteresis properties of a large-scale bypass MRF damper. The proposed PI model, with and without friction element, respectively, requiring minimal parameters of six and seven, accounts for current, displacement, and rate dependencies while maintaining simplicity.
• The proposed PI model is experimentally validated by characterizing the force-displacement and force-velocity responses of the designed MRF damper across a range of operating conditions within the training dataset. The model also accurately predicted the MRF damper’s hysteretic characteristics beyond the training dataset conditions. The model is further compared with the Bouc–Wen model, substantiating its overall benefits and accuracy in predicting damper behavior.
• Leveraging the simplicity, minimal parameter requirements, and analytic invertibility of PI models, the proposed PI model is positioned as a superior choice for facilitating controller design in smart structures employing MRF dampers. This underscores its practical applicability and contribution to the field of semi-active control systems.

The rest of the paper is outlined as follows. In Section 2, we explore the dynamic characterization of a bypass MRF damper. In section 3, the proposed PI model is developed. Section 4 discusses the main results and primary outcomes and Section 5 summarizes the main highlights.

## 2. Bypass MRF damper design and characterizations

**Figure 1.**
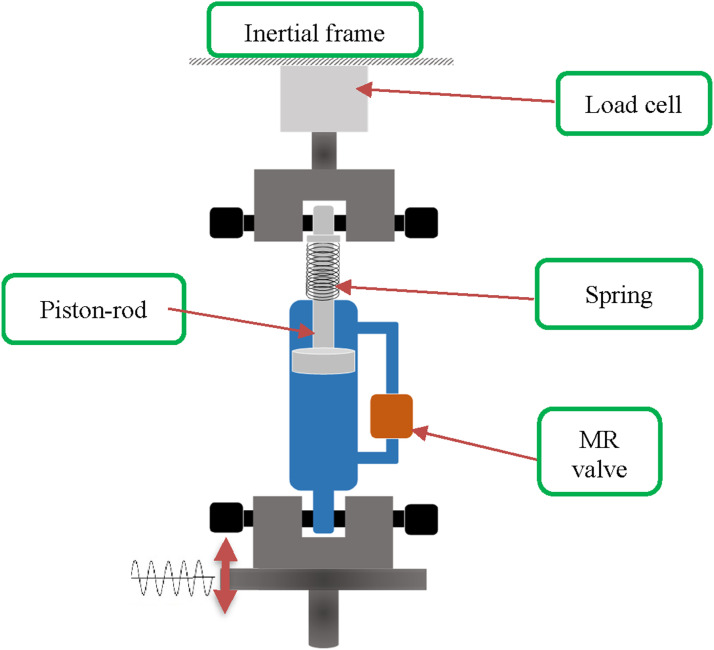
Schematic of the test setup.

A novel variable stiffness MRF damper with an annular-radial bypass valve was designed and prototyped for the study, as it has been reported in (Abdalaziz et al., 2023). Briefly, the MRF damper comprised of a main piston with piston rod inside a cylindrical housing, internal spring, and an external bypass magnetorheological valve (MRV), as schematically shown in Figure 1. The variations in the damping force are realized by controlling the magnetic field applied to the annular-radial bypass MRV, thereby producing high magnitude dynamic force and dynamic range. The annular-radial bypass MRV offers a relatively simple design for realizing high magnitude dynamic force and a broad dynamic range under field current ranging from 0 to 2 Amp. The proposed design with the external spring also offers variable stiffness. The damper design has thus been referred to as a variable stiffness and variable damping MRF damper. Furthermore, the bypass MRV offers enhanced heat dissipation property when compared to internal MRVs, particularly under high applied currents.

### 2.1. Dynamic characterization

An experiment was designed for dynamic characterizations of the prototyped bypass MRF damper, which has been described in (Abdalaziz et al., 2023). Briefly, the test setup consisted of a servo-hydraulic vibration exciter integrated within a Material Testing System (MTS), as schematically shown in Figure 1. The damper rod mounting was fixed to an inertial frame via a load cell, while the lower damper mount was fixed to the vibration exciter, as seen in Figure 1. The experiment was designed to measure the force-displacement and force-velocity characteristics of the damper under different harmonic excitations and field currents.

The heavy road and off-road vehicles generally exhibit vertical mode resonance in the vicinity of 2 Hz. The experimental characterizations were thus performed under low frequency excitations in the 0.5–4 Hz frequency (*f*) range, with displacement amplitude (*X*_0_) ranging from 1 to 2.5 mm, while the MRV coil current (*I*) was varied from 0 to 2 A. It should be noted that achieving a higher displacement amplitude, particularly at higher frequency and applied current was not possible due to constraints imposed by limitations in the test setup. The force and displacement signals were acquired using a data acquisition system (National Instrument) and analyzed in LABVIEW to obtain the force-displacement and force-velocity characteristics.

**Figure 2.**
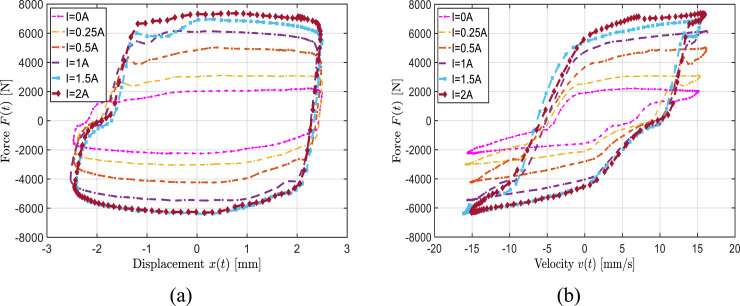
Variations in the measured force relative to the input displacement (a) and velocity (b) as a function of the current (*f* = 1 Hz, *X*_0_ = 2.5 mm).

As an example, Figure 2(a) illustrates the force-displacement response of the bypass MRF damper prototype for the entire range of applied current (0–2 A) under 2.5 mm amplitude excitation at 1 Hz. The results suggest increasing energy dissipation property of the damper with increase in the field current. A threefold increase in peak damping force is observed under the maximum current when compared to the passive mode, suggesting a wide band width of the damper. Increase in damping property with increasing current is also evident from increasing slope of the force-velocity curves, shown in Figure 2(b). These also exhibit considerable hysteresis apart from superior damping controllability. Similar trends were observed under other excitation conditions.

**Figure 3.**
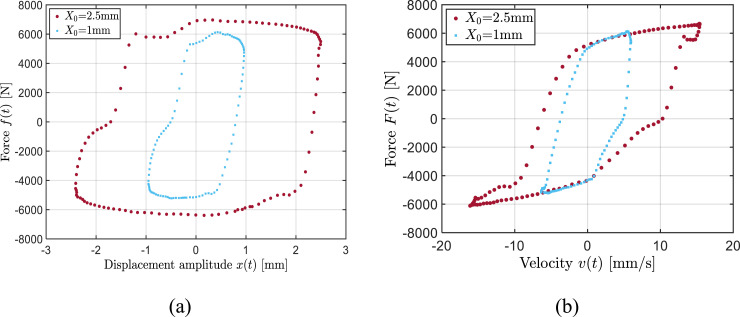
Influence of excitation amplitude on the measured force relative to the input displacement (a) and velocity (b) (*I* = 1.5 A, *f* = 1 Hz).

The results also show strong dependence of the damping force and the hysteresis on the damper displacement and velocity. This is further illustrated in Figure 3 considering two different displacement amplitudes (*X*_0_ = 1 mm and *X*_0_ = 2.5 mm) at a frequency of 1 Hz. The results are presented for an applied current of 1.5 A, as an example. Figure 3(a) exhibits significant increase in the dissipated energy, area bounded by the force-displacement hysteresis loop, with increase in excitation amplitude from 1 mm to 2.5 mm. The increase in damping characteristics with increasing displacement or velocity is also evident from the force-velocity curves shown in Figure 3(b). These trends were consistently observed under different field currents.

**Figure 4.**
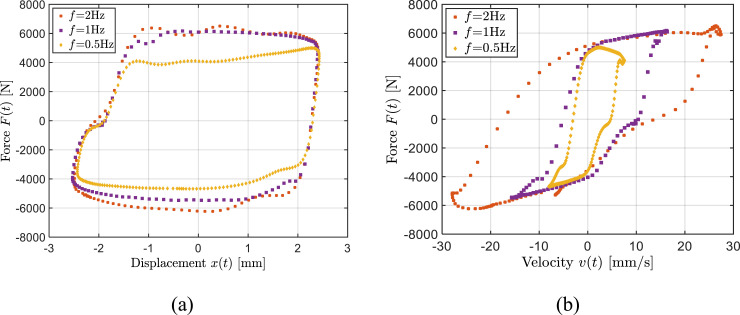
Influence of excitation frequency on the measured force relative to the input displacement (a) and velocity (b) (*I* = 0.5 A, *X*_0_ = 1 mm).

An increase in excitation frequency yields higher damper velocity and thereby higher damping force, as seen in Figure 4. The figure illustrates variations in the force-displacement and force-velocity characteristics of the bypass MRF damper as function of the excitation frequency. The results, as an example, are presented for a constant displacement amplitude of 1 mm and constant current of 0.5 A. The measured data exhibit an increase in the dissipative property of the damper with increase in the loading frequency. The results also show substantial reduction in the damping coefficient (slope of the force-velocity curve) as the velocity exceeds 5 mm/s, which is attributed to post-yield behavior of the MRF. The saturation tendency leading to reduction in effective damping coefficient at a higher velocity is clearly evident as the frequency increases to 1 Hz or 2 Hz. Such a property is desirable for vehicle suspensions in order to achieve enhanced ride performance (Debnath et al., 2021).

## 3. Model development

This section presents a novel stop-operators-based Prandtl-Ishlinskii model for describing the hysteretic dynamic properties of the bypass MRF damper as a function of the field current, excitation amplitude, and frequency.

### 3.1. Classical Prandtl-Ishiliskii hysteresis model

**Figure 5.**
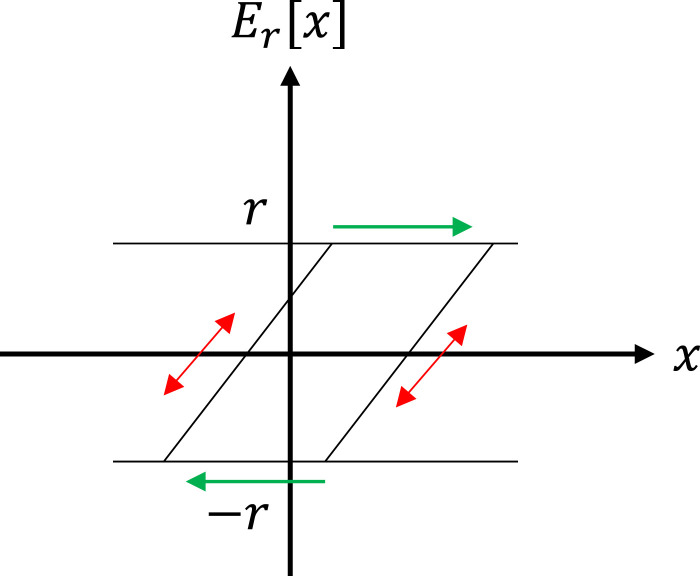
Stop hysteresis operator (Janaideh et al., 2009).

The PI model can be built using either the play or stop hysteresis operators. These operators are continuous hysteresis functions that can describe the input-output relationship for smart materials. The play and stop hysteresis operators exhibit counter-clockwise and clockwise hysteresis loops, respectively, and are characterized by the input x(t) and threshold r . The stop hysteresis operator, $E_{r}\left[x\right]\left(t\right)$ , shown in Figure 5, is employed to formulate the classical PI model as it corresponds to materials/systems showing clockwise hysteresis characteristics (e.g., magneto-active devices, MRF dampers) (Al Janaideh et al., 2011). The output corresponding to a given input can be achieved by a weighted superposition of the basic stop operators. For example, the force response $F_{PI}\left(t\right)$ of an MRF damper subject to an input displacement x(t) can be obtained as

(1) $$F_{PI}\left(t;x\right)=\overset{R}{\underset{0}{\int }}p\left(r\right)E_{r}\left[x\right]\left(t\right)dr≅\overset{N}{\underset{j=1}{\sum }}p\left(r_{j}\right)E_{r_{j}}\left[x\right]\left(t\right)$$

The function p(r) represents a density function that meets the condition p(r)>0 . The weights of the stop operators are given by $E_{r}\left[x\right]\left(t\right)$ , where R=+∞ is assumed for the sake of simplicity, meaning that p(r) will become negligible as r increases significantly. For any input $x\left(t\right)\in C_{m}\left[0,T\right]$ , the value of $E_{r_{j}}\left[x\right]\left(t\right)$ can be defined as (Janaideh et al., 2009)

(2) $$\left\{ \begin{array}{l} E_{r}\left[x\right]\left(t=0\right)=E_{r}\left(x\left(0\right)\right)\\ E_{r}\left[x\right]\left(t\right)=e_{r}\left(x\left(t\right)-x\left(t_{i}\right)+E_{r}\left[x\right]\left(t_{i}\right)\right);{\;t}_{i}<t\leq t_{i+1};\;0<i\leq N-1\\ e_{r}\left(x\right)=\min\;\left(r,\max\;\left(-r,x\right)\right) \end{array}\right.$$

It is assumed that the space of piecewise monotone continuous functions is denoted by $C_{m}\left[0,T\right]$ such that the input function x is monotone on the sub-intervals ( ${\;t}_{i},{\;t}_{i}+1),$ where [0,T] represents the time interval and ${\;t}_{i}$ denotes a point in time within this interval. Moreover, N is used to indicate the number of stop operators that are being considered, where j is used as an index for the stop operator (j=1,…,N) .

### 3.2. Formulation of the proposed modified PI model

The formulated PI model employs the stop operator $E_{r}\left[x\right]\left(t\right)$ and a density or weight function p(r) to characterize hysteresis nonlinearity. Stop hysteresis operators exhibit clockwise hysteresis loops, characterized by input x(t) and threshold r , describing the relationship between input x(t) and output $E_{r}\left[x\right]\left(t\right)$ . As mentioned before, stop operator is employed as it can represent materials/systems with clockwise hysteresis response characteristics such as MRF dampers. Combining weighted stop operators yields a symmetric output-input hysteresis response in practical applications, where a finite number of stop operators often suffice for modeling hysteresis. The classical PI model can then be constructed as a weighted sum of *N* stop operators

(3) $$F_{PI}\left(t;x\right)=\overset{N}{\underset{j=1}{\sum }}p\left(r_{j}\right)E_{r_{j}}\left[x\right]\left(t\right)$$

The classical PI model based on the stop operator is suitable for depicting symmetric force-displacement and force-velocity characteristics of MRF dampers. The PI model provides a versatile mathematical framework that permits the definition of model parameters, specifically the threshold and weighting functions. This flexibility allows for the incorporation of experimental factors such as input displacement, frequency, and applied current levels when determining these parameters. Mathematically it has been shown that the width of the hysteresis loop, associated with the stop operator, expands with increasing threshold $r_{j}$ (Al Janaideh et al., 2023), whereas mean slope of hysteresis loop has strong dependence on the selected weighting function (Al Janaideh et al., 2020). Therefore, threshold $\left(r_{j}\right)$ and density functions $\left(p\left(r_{j}\right)\right)$ selection should align with observed system behavior under various loading conditions and have physical relevance. Reported studies have proposed that a density function, taking the form of either an exponential or power function, adeptly can capture output saturation with increasing input (i.e., (Al Janaideh et al., 2008)). Experimental data presented in Figures 2–4 consistently indicated MRF damper force saturation with rising applied current, displacement, and frequency. Hence, a power function is selected as the density function, whereas the threshold function, $r_{j}$ , is presented as an exponential function of index j

(4) $$r_{j}=ae^{-bj};p\left(r_{j}\right)=r_{j}^{-k}=\alpha e^{\beta j};j=1,2,3,\ldots ,N$$

where a , b , α , and β are positive constants to be identified through the measured data. Even though the threshold function has been mostly presented as linear function of index j (Janaideh et al., 2009), the above-defined threshold function, $r_{j}$ , is presented as an exponential function of index j . It is because our analysis showed such exponential function better predict the output data. A similar threshold and density functions presented in equation (4), have also been reported in literature for predicting dynamic behavior of MR elastomers under dynamic loading (Dargahi et al., 2019). However, the threshold function presented in equation (4) lacks the ability to accommodate output force as a function of the applied current. To address this, the following modified threshold function is thus introduced to capture the field-dependency of the output force as

(5) $$r_{j}=\left(ae^{-bj}\right)\left(\frac{1}{1+ce^{-dI}}\right);\;j=1,2,3,\ldots ,N$$

where a,b,c, and d are positive constants that must be determined through the analysis of collected experimental data. The selected fractional-exponential function in equation (5) allows for the incorporation of the magnetic saturation phenomenon.

### 3.3. Model improvement

To further improve the simulation results, a friction element is added to the proposed modified PI model presented in equation (1) as

(6) $$F_{PI}\left(t;x,\dot{x}\right)=\overset{N}{\underset{j=1}{\sum }}p\left(r_{j}\right)E_{r_{j}}\left[x\right]\left(t\right)+F_{f}$$

(7) $$F_{f}=\gamma sign\left(\dot{x}\right)$$

where $F_{f}$ presents the friction element, according to the Coulomb friction law, and $\dot{x}$ and γ denote the input displacement-rate and the corresponding friction force, which is related to MR fluid yield stress at on-state condition where current is not zero. Under the off-state condition ( I=0A ), the friction element may physically signify the mechanical friction force produced by the motion of the piston, primarily originating from the bypass MRF damper seals positioned at various locations.

The parameters of the proposed PI models presented in equations (1) and (5) are identified through minimization of error between the model predicted and measure force-displacement and force-velocity characteristics of the damper. For this purpose, an error function, J(I) , is formulated as

(8) $$J\left(I,\Omega \right)=\overset{M_{k}}{\underset{k=1}{\sum }}\overset{N_{I}}{\underset{I=1}{\sum }}\left(F_{\mathrm{Exp}.}\left(t;I\right)-F_{PI}\left(t;I\right)\right)$$

where I is the applied current and Ω={a,b,c,d,α,β} is the parameters vector. The lower and upper bounds of the parameters were initially defined, and the minimization problem was solved in two sequential stages. In the first stage, a genetic algorithm (GA) was used to identify near global model parameters. The output of the GA was then imported to a gradient-based algorithm based on the sequential quadratic programming (SQP) in the second stage. The solutions obtained from SQP ensured the true global minima.

Furthermore, the minimization problem was solved by considering a subset of the measured data, denoted as the training data set. This subset comprised the measured force-displacement data for a fixed excitation ( $X_{0}=2.5\text{mm};f=1\text{Hz})$ but the entire range of field current (0, 0.25, 0.50, 1.0, 1.5, 2.0 A). This approach permitted not only the consideration of strong field-dependency of the damping force but also more efficient solution of the minimization problem. Table 1 summarized the identified model parameters on the basis of the training data subset.

**Table 1.** The identified parameters for the proposed PI model.

Without friction element							
a	b	c	d	α	β	*R*^2^(%)	
5.721	1.392	2.758	2.751	9.276	1.896	92.68	
With friction element							
a	b	c	d	α	β	γ(N)	*R*^2^(%)
0.711	1.17	3.074	2.84	355	1.4370	425	92.97

The validity of the model identified on the basis of the small subset of data is examined by comparing the model-predicted responses with the measured data acquired under all the excitations considered involving different excitation amplitudes and frequencies. The validation datasets correspond to 30 series of force-displacement characteristics of the bypass MRF damper, as presented in Table 2.

**Table 2.** The validation datasets for the PI models.

Displacement amplitude	1 mm			2.5 mm		
Frequency (Hz)	0.5	1.0	2.0	0.5	1.0	2.0
Current (A)	0, 0.25. 0.50, 1.0, 1.5, 2.0			0, 0.25. 0.50, 1.0, 1.5, 2.0		

### 3.4. Comparative analysis: PI model versus Bouc–Wen model

This section delves into a detailed comparison between the proposed PI model and the Bouc–Wen model, thereby allowing to further justify the advantages of the proposed PI model in predicting the MRF damper nonlinear and hysteretic behavior.

**Figure 6.**
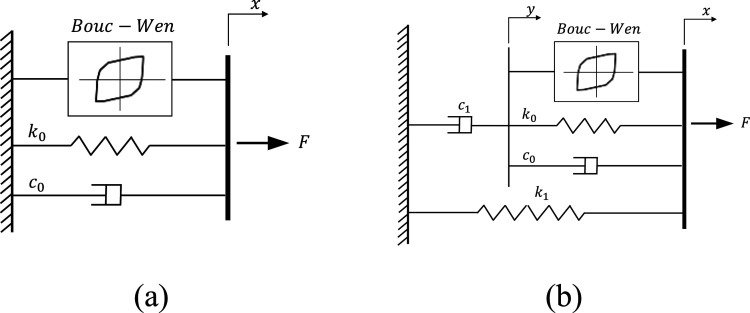
Different representations of Bouc–Wen model for MRF dampers: (a) simplified Bouc–Wen model; (b) standard Bouc–Wen model for MRF dampers (Spencer et al., 1997).

The initial presentation of the Bouc–Wen model dates back to 1976 when it was conceived by Robert Bouc and subsequently developed by Yi-Kwei Wen (Wen, 1976). The Bouc–Wen model, known for its versatility in demonstrating a broad spectrum of hysteretic behavior, is more intricate than the physical Bingham model (Shames, 1997; Stanway et al., 1987) and is commonly utilized to depict the nonlinear characteristics of MRF dampers (Spencer et al., 1997). Illustrated in Figure 6(a) is a schematic representation of this model in the simplest form. Using the Bouc–Wen model, the damping force may be expressed as

(9) $$F\left(t\right)=c_{0}\dot{x}+k_{0}\left(x-x_{0}\right)+\alpha z$$

where evolutionary variable, z , is given by

(10) $$\dot{z}=-\gamma .\left|\dot{x}\right|.z.\;{\left|z\right|}^{n-1}-\beta \dot{x}.{\left|z\right|}^{n}+A\dot{x}$$

The parameters α , β , γ , and A play a role in determining the roll-off transition between the pre-yield and post-yield regions (Spencer et al., 1997). On the other hand, n , in conjunction with the spring element ( $k_{0}$ ) and the initial displacement of the spring ( $x_{0}$ ), influences the shape of the hysteresis. The model configuration is depicted in Figure 6(a), illustrating a combination that includes a Bouc–Wen block in parallel with a spring ( $k_{0}$ ) and a viscous damper element ( $c_{0}$ ). In equation (9), $k_{0}$ may physically consider the presence of the accumulator within the damper, whereas $c_{0}$ indicates the linear viscous damper. The presented Bouc–Wen model in Figure 6(a), however, has shown limitations in accurately representing the roll-off and post-yield regions, particularly in the context of the force-velocity response (Sinjari, 2023; Spencer et al., 1997). In order to enhance the predictive capability of the damper response within this region, a more enhanced version of the system depicted in Figure 6(b) has been predominately employed as standard model in previous studies (Abdul Aziz et al., 2022; Jiang et al., 2023). In this case, the expression for the MRF damping force is given by (Spencer et al., 1997)

(11) $$F\left(t\right)=c_{0}\left(\dot{x}-\dot{y}\right)+k_{0}\left(x-y\right)+k_{1}\left(x-x_{0}\right)+\alpha z$$

where the evolutionary variable, z , is given by

(12) $$\dot{z}=-\gamma .\left|\dot{x}-\dot{y}\right|.z.\;{\left|z\right|}^{n-1}-\beta \left(\dot{x}-\dot{y}\right).{\left|z\right|}^{n}+A\left(\dot{x}-\dot{y}\right)$$

The internal displacement, y , shown in Figure 6(b), is determined by the following differential equation

(13) $$\dot{y}=\frac{1}{c_{0}-c_{1}}\left[c_{0}\dot{x}+k_{0}\left(x-y\right)+\alpha z\right]$$

The enhanced Bouc–Wen model illustrated in Figure 6(b) is an intricate analytical framework, featuring extra parameters ( $c_{1}$ and $k_{1}$ ) compared to the standard Bouc–Wen model presented in Figure 6(a). While viscous damping at higher velocities has been represented by $c_{0}$ , the damping coefficient $c_{1}$ in particular has been introduced to account for the damping effects of an additional dashpot, strategically incorporated to address the characteristic roll-off observed at lower velocities (Spencer et al., 1997). In section 4, we will elucidate and compare the results based on proposed PI models with the modified Bouc–Wen model presented in equations (11)–(13) against experimental data.

## 4. Results and discussions

In this section, the validity of the proposed PI model, both with and without friction element, is demonstrated by comparing the model-predicted responses with those obtained experimentally for the entire range of applied currents and mechanical loading conditions considered. Moreover, the effectiveness of the proposed PI model is further explored through a comparative analysis, comparing the PI model-predicted response with simulations results from the Bouc–Wen model.

### 4.1. Results and analysis on training datasets

**Figure 7.**
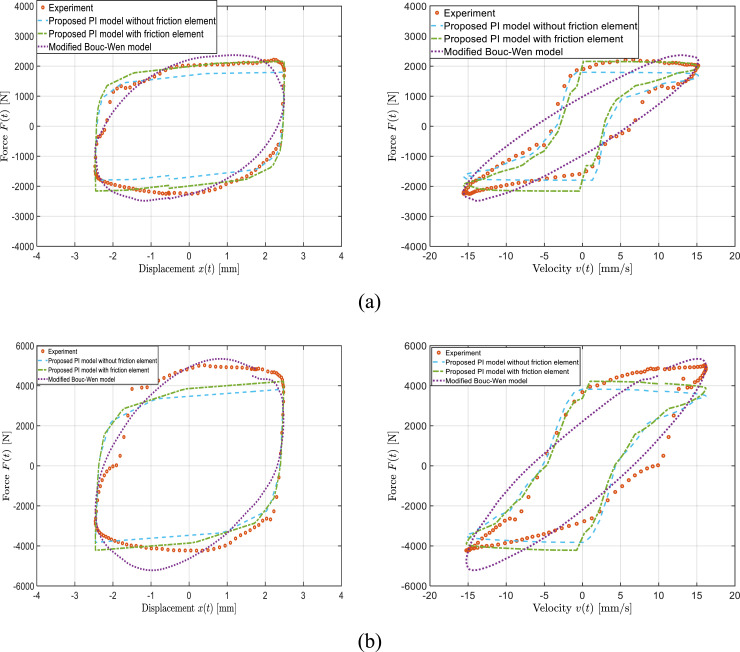
Comparison of model-predicted force-displacement and force-velocity responses of the bypass MRF damper with the experimental data considering two different field currents: (a) *I* = 0 A; and (b) *I* = 0.5 A $\left(X_{0}=2.5\;\text{mm};f=1\;\text{Hz}\right)$ .

Figure 7(a) and (b) show the comparisons of the PI model-predicted and measured force-displacement and force-velocity hysteresis responses of the MRF damper, as an example, under the applied current of 0 A and 0.5 A, respectively.

The results include PI model simulations with and without friction element. These figures also represent the performance of the presented modified Bouc–Wen model in predicting the force-displacement and force-velocity characteristics of the MRF damper. The results correspond to a loading frequency of 1 Hz and a displacement amplitude of 2.5 mm (data considered in the training data set). The results show reasonably good agreements between the dynamic behavior of the bypass MRF damper predicted by the proposed PI model without friction element and the measured responses considering the training dataset, although notable deviations are also evident. Such deviations are likely due to lack of consideration of the seal friction in the model. The PI model with friction element, however, slightly outperforms the PI model without friction model. The comparisons suggest that the proposed PI model, both with and without friction element, respectively, with only six and seven parameters presented in Table 1, are generally valid representations of the dynamic behavior of the bypass MRF damper under the given excitation conditions. Noted that results in terms of coefficient of determinations, R-square $\left(R^{2}\right)$ , quantitatively revealed slightly higher mean $R^{2}$ value of 92.97%, as presented in Table 3, for the PI model with friction element under the loading condition considered including all the applied currents, as compared to the PI model without friction element indicating mean R-square of 92.68%.

**Table 3.** Coefficients of determination ( $R^{2}$ ) for the proposed PI model without friction element at both training and validation datasets.

Frequency	Displacement amplitude(mm)	*I* = 0 A (%)	*I* = 0.25 A (%)	*I* = 0.5 A (%)	*I* = 1 A (%)	*I* = 1.5 A (%)	*I* = 2 A (%)	Mean (%)
Training datasets								
1 Hz	2.5	96.35	89.03	94.28	93.40	88.18	94.83	92.68
Validation datasets								
0.5 Hz	1	70.15	90.33	95.02	95.62	96.28	96.39	90.63
0.5 Hz	2.5	75.64	84.91	93.38	93.51	93.85	95.19	89.41
1 Hz	1	82.10	94.38	84.26	80.92	93.34	93.43	88.07
2 Hz	1	88.81	95.61	94.98	86.50	94.94	94.74	92.60
2 Hz	2.5	94.25	94.34	90.97	94.77	95.03	87.08	92.74

Figure 7(a) and (b) further revealed that Bouc–Wen model cannot completely capture the nonlinear force-velocity hysteresis characteristics of the MRF damper compared with proposed PI models with and without friction element. It should also be noted that the proposed PI model with and without friction element only use six and seven parameters for the entire range of loading conditioned considered as explained in next section, whereas the presented modified Bouc–Wen model does not have rate-dependency, displacement dependency, and current dependency. Thus, the Bouc–Wen model requires identification of 10 parameters ( $\left\{\alpha ,\beta ,\gamma ,n,A,x_{0},c_{0},c_{1},k_{0},k_{1}\right\}$ ) as presented in equation (10) through 14 for each loading condition. Our analysis showed that the Bouc–Wen model predictions are highly sensitive to variations in these parameters, and accurately identifying them through experimentation is a time-consuming and challenging task. The computational complexity of the Bouc–Wen model may limit its practicality in real-time applications or large-scale simulations. Moreover, while the proposed PI models permits consideration of magnetic saturation phenomenon, the Bouc–Wen model parameters don’t realize a clear physical interpretation, making it challenging to relate the Bouc–Wen model’s behavior to the underlying physical characteristics in the MRF damper behavior, including magnetic saturation, as well as current-, displacement-, and rate-dependency.

### 4.2. Results and analysis on validation datasets

**Figure 8.**
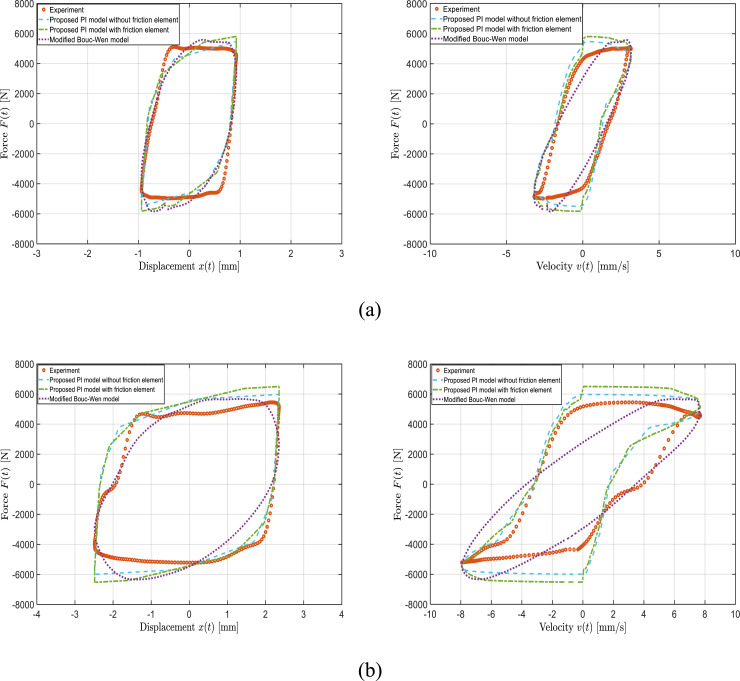
Comparison of model-predicted force-displacement and force-velocity responses of the bypass MRF damper with the measured data obtained under loading frequency of 0.5 Hz and applied current of 1.5 A: (a) $X_{0}$ = 1 mm (a); and (b) $X_{0}$ = 2.5 mm.

To scrutinize the proposed PI model’s performance more thoroughly, the predictability of the proposed PI model, both with and without friction element, was verified using the experimental validation datasets (Table 2) beyond the previously established training data set. Figure 8 compares the PI model-predicted force-displacement and force-velocity hysteresis responses of the bypass MRF damper with the measured data. The results, as an example, are presented for the applied current of 1.5 A and loading frequency of 0.5 Hz for different excitation amplitudes of 1 mm and 2.5 mm. Results show that both the proposed PI models can predict the dynamic response of the bypass MRF damper reasonably well under different displacement amplitudes.

**Figure 9.**
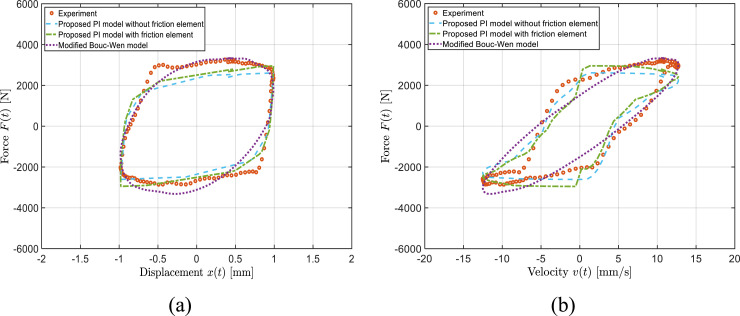
Comparison of model-predicted force-displacement (a) and force-velocity (b) responses of the bypass MRF damper with the measured data obtained under loading frequency of 2 Hz, applied current of 0.25 A, and displacement amplitude of $X_{0}$ = 1 mm.

**Figure 10.**
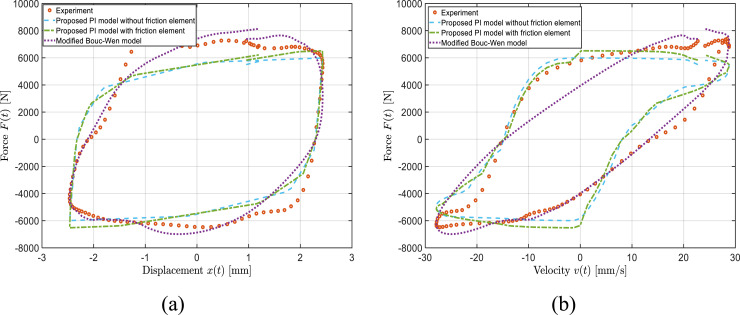
Comparison of model-predicted force-displacement (a) and force-velocity (b) responses of the bypass MRF damper with the measured data obtained under loading frequency of 2 Hz, applied current of 1.5 A, and displacement amplitude of $X_{0}$ = 2.5 mm.

Nonetheless, the PI model with friction element slightly better represents the complex curvature of the force-velocity properties. A relatively similar degree of prediction accuracy was obtained under other applied currents, loading frequencies and displacement amplitudes. For instance, Figures 9 and 10, as examples, present the PI model’s; predictions under displacement amplitude and current of 1 mm and 0.25 A as well as 2.5 mm and 1.5 A, respectively, under loading frequency of 2 Hz. Results presented in these figures are indicative of reasonably well prediction of both PI models and slightly better representation of the PI models with respect to the Bouc–Wen model in terms of hysteresis shapes in force-displacement and particularly force-velocity curves. The observed discrepancies between the PI models’ predictions and the experimental data, is partly due to the asymmetric characteristic (e.g., force-velocity curve in Figure 10(b)) of the designed variable stiffness and variable damping bypass MRF damper.

In addition to the visual representations, we conducted a quantitative analysis to evaluate the effectiveness of the proposed PI model, with and without friction element together with Bouc–Wen model across all the experimental conditions, including variations in applied current, loading frequency, and displacement amplitude. To quantify the accuracy of the proposed PI model’s predictions, we employed the, R-square $R^{2}$ .

The corresponding $R^{2}$ obtained for the predictions presented in Figure 10 are notably high for PI model without friction, PI model with friction, and Bouc–Wen model as 95.03%, 95.34%, and 96.82%, respectively. Similar values of determination coefficients were also obtained under different applied current conditions. Tables 3–5 summarize the coefficients of determinations of proposed PI model without friction, PI model with friction, and Bouc–Wen model, respectively, under the entire range of experimentally obtained force-displacement characteristic of the bypass MRF damper. These loading conditions include three frequencies (0.5 Hz, 1 Hz, and 2 Hz), two displacement amplitudes (1 mm, and 2.5 mm), and six applied currents (0 A, 0.25 A, 0.5 A, 1 A, 1.5 A, and 2 A), thereby resulting in a total of 36 experimental datasets. In most experimental scenarios including training and validation datasets, these $R^{2}$ values for PI model, with and without friction element, were greater than 90%, indicating that the proposed PI model can quite accurately capture the dynamic behavior of the bypass MRF damper. Tables 3 and 4 also revealed that when the applied current reaches its minimum value, the model’s performance generally diminishes, particularly at quasi-static regime (i.e., lower level of loading frequency of 0.5 Hz). Besides, the average coefficient of determination values for each loading condition considering all levels of current are also presented in Table 3 through Table 5. Considering the different mechanical loading conditions, the average coefficient of determination values considering different applied current, span from 88.07% to 92.74%, from 83.23% to 93.75%, and from 93.22% to 96.52%, respectively, for the PI model without friction, with friction, and Bouc–Wen model.

**Table 4.** Coefficients of determination ( $R^{2}$ ) for the proposed PI model with friction element at both training and validation datasets.

Frequency	Displacement amplitude (mm)	*I* = 0 A (%)	*I* = 0.25 A (%)	*I* = 0.5 A (%)	*I* = 1 A (%)	*I* = 1.5 A (%)	*I* = 2 A (%)	Mean (%)
Training datasets								
1 Hz	2.5	95.53	90.13	**94.87**	93.30	**88.98**	**94.99**	**92.97**
Validation datasets								
0.5 Hz	1	36.89	82.50	92.91	95.00	95.82	96.27	83.23
0.5 Hz	2.5	61.90	79.69	90.76	91.62	92.33	94.29	85.10
1 Hz	1	76.72	**94.42**	**85.77**	**83.99**	93.29	93.07	87.88
2 Hz	1	**89.23**	**95.79**	**96.70**	**88.90**	**95.96**	95.62	93.70
2 Hz	2.5	**95.27**	**95.80**	**92.98**	**95.26**	**95.34**	**87.84**	93.75

**Table 5.** Coefficients of determination ( $R^{2}$ ) for the modified Bouc–Wen model across all the conditions considered.

Frequency (Hz)	Displacement amplitude (mm)	*I* = 0 A (%)	*I* = 0.25 A (%)	*I* = 0.5 A (%)	*I* = 1 A (%)	*I* = 1.5 A (%)	*I* = 2 A (%)	Mean (%)
0.5	1	95.46	95.66	95.08	94.67	96.57	95.31	95.46
0.5	2.5	91.55	93.74	94.11	93.18	93.58	93.15	93.22
1	1	93.83	95.77	96.11	96.57	96.16	96.73	95.86
1	2.5	95.39	95.20	95.70	94.03	95.03	95.43	95.13
2	1	95.05	95.79	96.81	97.12	95.90	96.32	96.17
2	2.5	95.13	96.81	96.86	96.70	96.82	96.81	96.52

Moreover, average of mean $R^{2}$ values across all the loading condition considered, were obtained as 91.02%, 89.44%, and 95.39% for the PI model without friction, with friction, and Bouc–Wen model as presented in Table 3 through Table 5. Nonetheless, in half of the experimental cases, as highlighted in bold in Table 4, the proposed PI model with friction element outperformed PI model without friction. More specifically, analysis of Tables 3 and 4 further inferred that the PI model with the friction element quite consistently exhibited superior performance compared to the model without friction, especially as the frequency surpassed 0.5 Hz. It is in part due to the significant contribution of friction force at higher velocities.

Furthermore, results in Tables 3 and 4 demonstrate that the proposed PI model, both with and without friction element can even extend its predictions to beyond the range of the training experimental dataset, maintaining reasonable accuracy levels. It is also worth mentioning that the PI model with and without friction element only require six and seven parameters, respectively, to predict the dynamic behavior of the bypass MRF damper, whereas the Bouc-–Wen model requires 10 parameters for each set of loading conditions, consequently requiring the identification of 360 (10 × 36) parameters. Thus, the Bouc–Wen model may exhibit limitations, particularly in terms of its suitability for real-time applications or large-scale simulations. These limitations arise from its sensitivity to parameter variation, computational complexity, and a lack of clear physical interpretability. It should similarly be mentioned that the proposed PI model, both with and without friction element, has limitation in predicting MRF damper characteristics under relatively lower level of applied current and loading frequencies, as can be seen in Tables 3 and 4. All in all, leveraging the simplicity, minimal parameter requirements, relatively high R-square values, and the analytic invertibility inherent in PI models, the proposed PI model stands out as a superior option for predicting the dynamic characteristics MRF dampers.

The results presented in this study suggest that the proposed PI model has the potential to be a useful tool for predicting the hysteresis behavior of the MRF damper under various operating magnetic and mechanical loading conditions. The suggested model is also mathematically invertible, making it a viable candidate for designing controllers for intelligent structures that incorporate MRF dampers.

## 5. Conclusions

In this paper, a novel Prandtl-Ishlinskii (PI) model with reduced number of parameters is formulated using a set of training dataset to predict the dynamic behavior of a prototyped bypass MRF damper under a wide range of applied currents and mechanical loading conditions. The proposed PI model, with and without the friction element, has been rigorously validated across a broad range of applied current, loading frequency, and displacement amplitude, demonstrating its effectiveness. The effectiveness of the PI model was further assessed through a comparative analysis with simulations from a modified Bouc–Wen model. Extended evaluations with validation datasets revealed that the PI models’ versatility, demonstrating reasonable prediction accuracy under varying applied current, frequencies, and displacement amplitudes. Quantitative assessments showed that the average coefficient of determination (*R*^2^) values considering different applied current, spans from 88.07% to 92.74%, from 83.23% to 93.75%, and from 93.22% to 96.52%, respectively, for the PI model without friction, with friction, and the modified Bouc–Wen model. The PI model, when equipped with the friction element, generally exhibited superior performance compared to the PI model lacking friction, when loading frequency surpassed 0.5 Hz. Despite the Bouc–Wen model’s relative efficacy, its complexity, computational cost, sensitivity to parameters, and lack of clear physical interpretability pose significant challenges. The PI models, with and without friction element, respectively, requiring minimal parameters of six and seven, exhibit interpolating and extrapolating (beyond the training dataset) predictive capabilities, in contrast to the current-, frequency-, and displacement-independent Bouc–Wen model, which demands a significantly larger parameter set (10) for set of each loading condition. The proposed PI model can be served as a superior choice for predicting the dynamic characteristics of MRF dampers, given its simplicity, minimal parameters, and high $R^{2}$ values, while offering current-, frequency-, and displacement-dependencies. This research established the PI model as a valuable tool for comprehending and forecasting the hysteresis behavior of MRF dampers, presenting potential applications in the development of controllers for intelligent structures that integrate these dampers.
